# Mr. Cuming, on Digitalis in Pneumonia

**Published:** 1804-08-01

**Authors:** Ralph Cuming

**Affiliations:** Romsey, Hants


### *To the Editors of the Medical and Physical Journal.*

GENTLEMEN,

A Delicate female, ætat. 25, sent for me on the 19th of May, who I found labouring under a complication of diseases; she had been for some time subject to dysentery, and now was seized with a more formidable and alarming complaint, namely, pneumonia; inspiration was attended with the most acute stitch of her right side, and she was obliged to be propped up in bed, not being able to endure a recumbent posture; her pulse was full and frequent, countenance flushed; and though she was much debilitated by the alvine discharge, phlebotomy appeared to be the only remedy calculated to afford relief in this dilemma; I therefore took from her arm twelve ounces of blood, sent her a refrigerant medicine, and left the strictest injunctions respecting her regimen.

On the following day, the smallest amendment was not perceptible; the stitch, purging and pyrexia continued with unabated violence. I now determined to give the *tinct. digitalis* a fair trial, but thought I should not be justified by leaving my patient in so much distress, without again availing myself of the advantage of another bleeding; I accordingly drew off eight ounces more, and sent her a blister, to be applied to the part, with these draughts: ℞. Spt. æth. nitrosi ʒj. tinct. digitalis gtt. 40, aq. puræ ℥ij. M. ft. haust. mitte no. ij. capt. j. bis de die. In the evening I visited her, and sent another nitrous draught, omitting the digitalis, not wishing to push it any farther the first day.

On the 21st, I found her in a recumbent posture, inclining to her right side; she said the smallest movement from the present position, occasioned inexpressible torture; her countenance was less flushed, and her skin felt moist, but she observed that the stitch was as bad as ever; she neither could bear any one to move her, nor could she alter her position. Let me ask any of my medical brethren, if a repetition of bleeding was not plainly indicated, according to the received opinion of all authors; and such treatment would have been considered sound practice, and would most assuredly have been adopted by any one, whose experience had not taught him, that there yet remained a remedy, which surpassed the most sanguine expectations that could have been conceived of it, in the cure of phthisis pulmonalis; and reasoning ipso facto, about the modus operandi ejusdem medicaminis, in this disease, would be encouraged to hope for success from its administration in pneumonia also. As her purging and tenesmus still remained, I this day increased the digitalis to gtt. 100, (omitting the spt. æther nitros.) which she took at three different periods, that is, to gtt. 33 to a dose.

The 22d, she was able to alter her position, and observed the air descended further into her lungs, though a hasty inspiration could not have been endured. Her pulse had sunk considerably; her face bore evident marks of anasarca, its enlargement and puffy feel being noticed by herself and every one who saw her. I had sufficient cause to congratulate myself on the resolution I formed, of abandoning the use of the lancet, although the last blood I took away, exhibited that cup-like appearance so frequently observed, having the edge of the crassamentum curled in, and its whole surface covered with a very thick crust of coagulable lymph. The same quantity of digitalis was continued this day.

The 23d, she was able to set up in bed, respiration became easy, and she only complained of soreness in the part; her pulse was now about 60, and as the danger from inflammation vanished, I began to direct my attention to the state of her bowels; the aliment passed off almost as fast as it was received; pain and tenesmus she still suffered greatly from. ℞. Tinct. opii gtt. 40, digital. gtt. 30, aq. puræ ℥ij. M. ft. haust. horâ decubitûs sumend. I have an idea that digitalis counteracts the stimulant effects of opium, which was my reason for combining them in this instance.

The 24th, she passed a tolerable night, and to day took the two following draughts. ℞. Tinct. opii, gtt. 30; digit. gtt. 20, aq. puræ ℥ij. M. ft. haust. A. M. et horâ somni.

The 25th, her motions were less frequent. I now omitted the digitalis, and gave her pulv. ipec. comp. gr. viij ter de die; this medicine by the assistance of cret. ppt. occasionally conjoined, with a generous diet, completed the cure.

On the 4th of June she was free from every complaint, debility excepted, continuing to regain lost stamina. I forgot to mention that this woman was suckling a stout boy, twelve months old, which I prevailed upon her to wean.

Another instance of pulmonic inflammation occurred to me the other day, occasioned by living freely, and blowing too much upon musical instruments. My patient was a young young miller, attached to a volunteer corps; and in this case the digitalis was crowned with the same success; in less than twenty-four hours, his pulse, which had been at 120, was reduced to 60; his countenance though highly crimsoned, by the same mean, was changed to its natural aspect.

I have to observe, when we are called in to visit patients in this acute and dangerous disease; that it would be improper to trust entirely to the effects of digitalis, because some time will elapse before it exerts its influence in the body, so as to check the undue impetus of the blood; but as no time can consistently with the safety of the patient be lost, I conceive it ought to be administered immediately after the first bleeding, in a quantity proportioned to the urgency of the case, and it will frequently happen that a second bleeding may be requisite, before this potent sedative has effected our purpose.

I imagine it operates chiefly on the nervous system, and is indued with the peculiar property of restraining morbid excitability, or in reducing the increased mobility of the vital principle to its proper level and healthy standard. How often the lancet has been, and is still, used in pneumonic cases, is too well known to every practitioner; and who is there that has not witnessed the melancholy effects emanating from this mode of depletion? pining *atrophy!* lingering *dropsy!* and a variety of evils, occurring so frequently, as to render a particular account of them in this place quite unnecessary, occasioned entirely by robbing the body of that *pabulum,* which is the sine quâ non of its existence.

I think it fair to conclude, that my female patient, who was nearly worn thread-bare by disease, previously to her having been seized with pneumonia, would in all probability have died dropsical, if the lancet instead of digitalis had been solely confided in. Then, what inferences may be deduced from this conclusion? The happiest presages may surely be formed of its salutary effects, when employed as frequently as its virtues demand it should be.

This medicine admits of more extensive application than many may suppose; in *asthma,* it will afford great relief. I had a patient the other day, to whom I administered the remedies generally used on these occasions, without deriving any advantage from them; but as soon as he took this medicine his health improved daily. It is in this complaint as well as many others, that theory and practice are at variance, yet I must say, according to the opinion I

have formed of it, that the Materia Medica cannot boast of a medicine, which does in a general way more effectually fulfil every intention, considered in a pathological point of view. Some time ago, I used it in ascites and anasarca: A man, aged fifty-five, who laboured under an intermittent a considerable time, in Essex, was so much reduced by it when he applied to me, that I formed a very unfavourable prognosis of him; the cells of the adipose membrane from head to foot were filled with water, and the *abdomen* so much distended that it did not contain less than three gallons of fluid. His intermittent, a quotidian, was easily cured by *zinc. vitriol.* (a medicine which I took occasion to recommend the other day in your Journal) gr. v. indies; after which, he took squills, calomel, &c. rubbing in ung. hydr. fort. during this course. The squills, though given in liberal doses, were of no avail in discharging the water; but as soon as digitalis was employed, the extravasated fluid soon made its exit, in copious quantities, and the man is now enjoying good health, free from any appearance of fresh accumulation.

As the human mind is ever liable to run into extremes, outstripping the boundaries of calm and dispassionate reason; I wish to observe, that the high opinion which *I* entertain of this remedy, is founded on the unerring test of experience, the guide and polar star of every science, and which is the only rational way by which we can hope to attain to any mode of improvement in Physic, that in so many instances is veiled by a mist, through which the brightest beams of human understanding cannot penetrate.

After having most faithfully represented cases in support of this excellent medicine, where its effects must appear so evident and striking, as to defy and put keen edged casuistry to the blush; need I fear the snarls of the incredulous, or care for the distempered remarks of the idle disputant, who more for the sake of displaying his controversial powers, than benefitting society, so frequently takes shelter under a borrowed name, and thus cowardly, when secure behind the bastion of obscurity, lets fly his envenomed shafts.

There are men whose timidity prevents them from venturing out of the beaten track of their predecessors; it is not from *such* that we can expect improvements, particularly in Physic, where so much remains to be done; for as a Professor properly observes to his pupils, "Your present instructions are only intended as a foundation on which you are to build." As if conscious of innumerable difficulties, difficulties, which must ever remain insurmountable, should the mind not be suffered to soar beyond the narrow limits of scholastic dogmas.

> De tuo ipsius studio conjecturam ceperis
> An veritas aut perfidia hanc mentem gubernat.

I shall conclude with a borrowed phrase reversed, mehercule digitalis sedat! I am, &c.

